# Ultrasound − assisted sodium alginate coating pretreatment integrated with advanced drying technologies: A novel strategy to optimize drying behavior, energy consumption, physicochemical quality, and sensory attributes of *Zanthoxylum bungeanum*

**DOI:** 10.1016/j.ultsonch.2026.107901

**Published:** 2026-05-22

**Authors:** Zepeng Zang, Xiaopeng Huang, Guojun Ma, Qiaozhu Zhao, Yanrui Xu, Bowen Wu, Fangxin Wan

**Affiliations:** a College of Mechanical and Electrical Engineering, Gansu Agricultural University, Lanzhou 730070, China; b College of Life Sciences, Shaanxi Normal University, Xi’an 710119, China

**Keywords:** *Zanthoxylumbungeanum*, Multi−frequency ultrasound, US−SA coating pretreatment, Advanced drying technologies, Energy consumption

## Abstract

To address the complex moisture migration pathways, limited process adaptability, and pronounced quality deterioration during the drying of *Zanthoxylum bungeanum*, this study introduced an ultrasound–sodium alginate (US − SA) pretreatment and coupled it with multiple advanced drying technologies, including multi − frequency ultrasound − vacuum far − infrared drying (MFUS − FIRD), MFUS − HAD, radio frequency vacuum drying (RFVD), microwave vacuum drying (MVD), far–infrared vacuum drying (FIRD), and hot air drying (HAD). The drying kinetics, energy consumption, sensory attributes, and quality evolution of *Zanthoxylum bungeanum* under different processes were systematically compared. The results indicated that (US − SA) − MVD achieved the highest drying efficiency (260 min) and the lowest energy consumption (33.25 kW·h·k^g−1^), whereas its quality retention was limited. In contrast, *Zanthoxylum bungeanum* treated by (US − SA)−(MFUS − FIRD) exhibited significantly higher retention of total phenolics, total flavonoids, antioxidant capacity (DPPH = 63.44%, ABTS = 95.47 mg Trolox/g, and FRAP = 65.48 mg Fe^2+^/g,), total alkaloids, total amides, and hydroxy-α-sanshool, together with superior color preservation (ΔE = 6.56, BI = 45.85, C = 15.63) and a well-maintained, uniformly porous microstructure. GC − MS and electronic nose analyses demonstrated that (US − SA)−(MFUS − FIRD) favored the retention of terpenes, terpene alcohols, and esters, while suppressing oxidative degradation and off-flavor formation; the responses on W1W, W1S, and W3S sensors reached 27.736, 5.670, and 1.910, respectively, significantly exceeding those of other dehydration methods. Sensory evaluation revealed that (US − SA)−(MFUS − FIRD) and (US − SA)−(MFUS − HAD) samples exhibited higher numbing sensation, spiciness, fresh aroma, and overall acceptability, with lower bitterness and off-odors. Hierarchical clustering heat map and PCA further confirmed the pronounced advantage of (US − SA)−(MFUS − FIRD) in the multidimensional quality space. This study elucidates the quality formation mechanism of *Zanthoxylum bungeanum* under ultrasound − coating pretreatment coupled with advanced drying technologies, providing a theoretical basis and technical reference for its low − energy consumption and high–quality processing. Future research should focus on industrial-scale applications and intelligent regulation of ultrasound-assisted hybrid drying systems.

## Highlights

• (US − SA) − MVD treatment exhibited the highest drying rate but compromised product quality.
• US − SA pretreatment enhances quality and volatile compounds in Zanthoxylum bungeanum.
• MFUS and US–SA pretreatment positively contributed to microstructure preservation.
• (US − SA)−(MFUS − FIRD) treatment maintained higher antioxidant capacity and better color.
• The samples treated with (US − SA)−(MFUS − FIRD) achieved the highest overall acceptance.

## 1 Introduction

*Zanthoxylum bungeanum* Maxim., the mature fruit of a Rutaceae species, is a typical food − medicine homologous plant and is acclaimed as the “King of Oriental Spices” owing to its distinctive aroma and tingling sensation [1], [2]. As a key component of traditional dietary culture and medicinal systems, *Zanthoxylum bungeanum* holds an irreplaceable position in Chinese culinary seasoning and is also widely utilized in Japan, Korea, India, and in certain regions of Europe and North America [3], [4]. In recent years, with the growing global demand for natural seasonings and health − oriented foods, its economic and industrial value in international markets has increased markedly. The pericarp of *Zanthoxylum bungeanum* is rich in volatile oils, alkaloids, amides, polyphenols, flavonoids, and unsaturated fatty acids [5], which impart its characteristic aroma and numbing sensation and exhibit notable pharmacological activities, including antioxidant, antimicrobial, anti − inflammatory, and neuroprotective effects [6], [7]. Consequently, *Zanthoxylum bungeanum* possesses considerable economic and application value in both the food industry and health − related sectors. However, the fruit displays high moisture content, vigorous respiration, and fragile cellular structures at maturity and, if not promptly processed, readily undergoes browning, flavor deterioration, and degradation of bioactive constituents due to enzymatic reactions, oxidation, and microbial contamination, thereby compromising quality and storage stability [8], [9]. Thus, the development of efficient drying technologies is critical for large–scale processing and value–added utilization of *Zanthoxylum bungeanum*, as well as for prolonging its shelf life, preserving physicochemical attributes, and enhancing product value.

As one of the most critical stages in the processing of *Zanthoxylum bungeanum*, drying reduces moisture content and suppresses microbial growth, thereby effectively delaying enzymatic browning, lipid oxidation, and the degradation of aroma − active compounds, ultimately enhancing storage stability and shelf life [10], [11]. In addition, the drying process, while ensuring product safety, plays a decisive role in determining the color, aromatic profile, textural attributes, and retention of bioactive constituents in *Zanthoxylum bungeanum*. At present, drying of *Zanthoxylum bungeanum* primarily relies on traditional methods such as sun drying and hot air drying (HAD). Sun drying depends on natural conditions and, despite its low cost, is constrained by weather and hygienic factors, often resulting in the loss of volatile compounds and fluctuations in quality [12]. By contrast, HAD is widely used in industrial production due to its simple equipment and broad applicability, yet its long processing time and high energy consumption, coupled with the susceptibility of volatile oils, amide compounds, and the pericarp to degradation and browning under high temperatures, adversely affect physicochemical and sensory quality [13]. Furthermore, during conventional hot-air drying, the mismatch between internal moisture diffusion and surface evaporation rates tends to induce case hardening and structural shrinkage, thereby further impeding moisture migration and exacerbating physicochemical and sensory quality deterioration. These limitations render traditional drying methods insufficient to meet the integrated requirements of modern food processing for high quality, low energy consumption, and high efficiency. To overcome the drawbacks of conventional drying and in response to growing consumer demand for high − quality flavorful foods, modern drying technologies have been increasingly applied in the processing of *Zanthoxylum bungeanum*, offering improved energy efficiency and enhanced quality retention as effective pathways to increase product value. Currently, widely applied modern drying techniques include ultrasound − assisted drying [14], microwave drying [15], far − infrared drying [16], freeze drying [17], and radio frequency heating [18].

Vacuum far − infrared drying (FIRD) integrates the advantages of low − pressure conditions and far − infrared radiation. Under vacuum, the boiling point of water decreases, accelerating evaporation, while the low − oxygen environment effectively suppresses oxidation and enzymatic browning. Far − infrared radiation exhibits strong penetration, enabling faster migration of internal moisture toward the surface and preventing quality degradation associated with prolonged high − temperature exposure [19]. Incorporating multi − frequency ultrasound (MFUS) into FIRD establishes a multi − frequency ultrasound–vacuum far–infrared drying (MFUS − FIRD) system, in which the combined energy fields further enhance heat and mass transfer. The cavitation and microstreaming generated by ultrasound weaken the boundary − layer resistance on the material surface and increase cellular porosity and the number of capillary channels, thereby markedly promoting outward moisture diffusion [20], [21]. Compared with single − frequency ultrasound (SFUS), MFUS provides more uniform acoustic − field distribution and higher energy utilization, reducing the adverse effects of localized energy concentration and improving drying stability [22]. When MFUS is coupled with vacuum conditions and far − infrared radiation, moisture − migration pathways become more unobstructed, drying rates increase substantially, and losses of bioactive substances and volatile aroma compounds are effectively controlled, rendering the physicochemical properties and sensory quality of the product closer to those of fresh material. Studies have demonstrated that this approach significantly improves rehydration capacity, texture, and aroma retention and is therefore regarded as an important direction for enhancing both drying efficiency and quality preservation in agricultural products [23], [24]. Building on this, MFUS has also been applied in HAD, forming multi − frequency ultrasound − assisted hot air drying (MFUS − HAD). Through the more uniform acoustic field and energy transmission associated with MFUS, this technique alleviates the uneven heating and dehydration commonly observed in conventional hot air drying and reduces energy consumption while shortening the drying cycle [25]. Microwave vacuum drying (MVD) and radio frequency vacuum drying (RFVD) are both electromagnetic-field-based technologies in which electromagnetic waves interact with polar molecules within the material, inducing rapid molecular vibration and frictional heating. These effects enable volumetric heating, markedly shorten drying time, and reduce conductive heat loss [26], [27]. MVD operates at higher frequencies and offers rapid heating, yet the nonuniform distribution of the electromagnetic field may cause localized overheating, leading to degradation of thermolabile compounds and impairments in color and flavor; thus, intermittent operation or rotating structures are often employed to improve uniformity. RFVD, by contrast, utilizes the longer wavelength and stronger penetration of radio frequency waves under low − pressure conditions, facilitating internal water vaporization and migration, improving heat and mass transfer efficiency, and preserving bioactive constituents and sensory quality at relatively low temperatures, making it well suited for thermally sensitive agricultural products [28], [29]. Although the aforementioned modern drying technologies have demonstrated considerable potential in improving drying efficiency, systematic comparisons and in-depth investigations into their effects on the retention mechanisms of volatile flavor compounds, numbing-active constituents, and bioactive components in *Zanthoxylum bungeanum* remain limited.

Alongside advances in drying technologies, pretreatment strategies also play a crucial role in preserving the quality of *Zanthoxylum bungeanum*. In recent years, edible coatings have emerged as an important approach for improving drying quality due to their safety, biodegradability, and environmental compatibility [30]. Sodium alginate (SA), a natural polysaccharide with favorable film forming capacity, moderate gas permeability, and excellent moisture − retention properties, can form a uniform protective barrier on the surface of *Zanthoxylum bungeanum*, thereby slowing moisture migration–induced pericarp cracking and reducing the loss of volatile aroma compounds and thermolabile bioactive constituents [31], [32]. Furthermore, the coating can inhibit oxygen diffusion and browning reactions, delaying quality deterioration and enhancing storage stability. When combined with ultrasound, acoustic cavitation facilitates the penetration and uniform distribution of the coating solution across tissue surfaces, improving coating adhesion and compactness and further strengthening protection of volatile and heat − sensitive compounds [33]. This synergistic approach not only improves heat and mass transfer conditions but also provides a new technical pathway for enhancing the physicochemical and sensory quality of dried *Zanthoxylum bungeanum*.

Until now, most existing studies focus on either a single drying technique or a single pretreatment measure, with insufficient attention to the integrated optimization of drying efficiency and quality preservation in *Zanthoxylum bungeanum*, particularly regarding aroma compound volatilization, color deterioration, and the degradation of bioactive substances. Moreover, research on the synergistic mechanisms between pretreatments and drying methods is still limited, constraining further process optimization. Therefore, it is necessary to develop an integrated drying system that simultaneously achieves efficient dehydration, low energy consumption, and effective quality preservation, while elucidating its underlying quality regulation mechanisms. Based on this, the present study employed an ultrasound–sodium alginate composite pretreatment coupled with advanced drying technologies to systematically evaluate their effects on the drying characteristics, energy consumption, and quality formation of *Zanthoxylum bungeanum*. This study innovatively proposed an ultrasound–edible coating–hybrid drying synergistic system and further elucidated its synergistic enhancement mechanisms in flavor retention, bioactive compound preservation, and energy consumption regulation. The research provides valuable theoretical guidance and process references for the development of high − quality *Zanthoxylum bungeanum* and energy − efficient, sustainable drying technologies.

## 2 Materials and methods

### 2.1 Experimental materials

The *Zanthoxylum bungeanum* used in this study was sourced from Maiji District, Tianshui City, Gansu Province. Samples with high maturity, uniform coloration, intact pericarps, and consistent size were selected as the experimental material. After preliminary impurity removal and selection, the initial moisture content was measured as 63.90 ± 1.0% (w.b.). To reduce color loss during the drying process, all samples were subjected to a color protection treatment (the composite color protecting solution was prepared from 0.5% citric acid, 0.5% ascorbic acid, and 0.5% L − cysteine), followed by subsequent ultrasound–sodium alginate (US − SA) composite pretreatment and different drying experiments.

### 2.2 **US** − **SA coating pretreatment**

An appropriate amount of SA powder was dissolved to prepare a 0.5% (w/v) aqueous solution, which was then slowly added to deionized water at 40°C and stirred for 30 min using a magnetic stirrer until fully dissolved to form a homogeneous SA solution. Subsequently, freshly harvested *Zanthoxylum bungeanum*, cleaned and freed of impurities, was immediately immersed in the prepared 0.5% (w/v) SA solution for 2 min to ensure uniform surface coverage. The samples were then placed in an ultrasonic bath (ultrasound frequency 40 kHz, ultrasound power 120 W) and treated at 25 ± 1°C for 15 min, with temperature controlled by a circulating cooling system. After treatment, the samples were removed and drained to eliminate residual surface solution for subsequent use.

### 2.3 Drying experimentals

#### 2.3.1 Control experiments

**Fig. 1:**
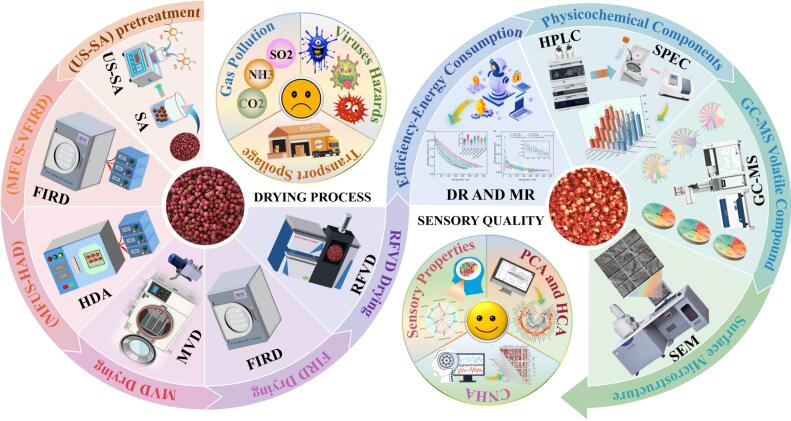
Experimental flow chart of US–SA pretreatment combined with different drying processes for *Zanthoxylum bungeanum*.

The overall experimental workflow for *Zanthoxylum bungeanum* subjected to US–SA pretreatment in combination with different drying techniques is illustrated in Fig. 1.

Control experiment 1 ((US − SA) − HAD): *Zanthoxylum bungeanum* samples (300 ± 1.0 g) pretreated by US–SA were placed on stainless − steel trays in a hot air dryer for dehydration. Based on preliminary trials, the drying temperature was set at 55°C, the airflow velocity was maintained at 1.0 m/s, and the material layer thickness was approximately 10 mm. During drying, the samples were removed every 10 min for weighing, with each weighing completed within 15 s. To ensure uniform heating, the samples were turned every 20 min. Drying was terminated when the moisture content of *Zanthoxylum bungeanum* reached 10% (wet basis), and each experiment was conducted in triplicate (same below).

**Fig. 2:**
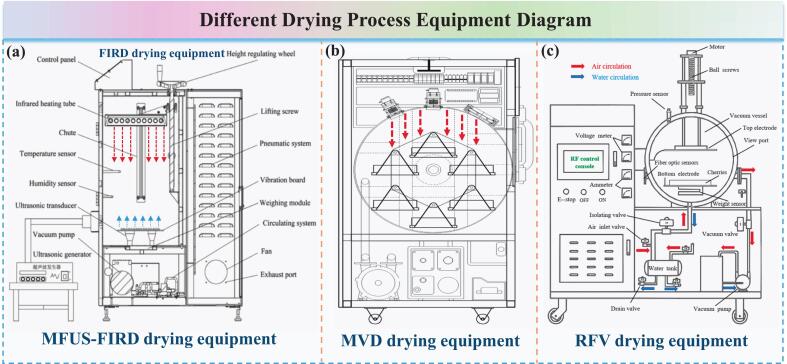
Typical advanced drying equipment used in the experiments. (a) MFUS–FIRD equipment; (b) MVD equipment; (c) RFVD equipment.

Control experiment 2 (MFUS − FIRD): Fresh *Zanthoxylum bungeanum* (300 ± 1.0 g, without pretreatment) was placed in the multi − frequency ultrasound–vacuum far − infrared drying (MFUS − FIRD) system for dehydration (Fig. 2(a)), with a material layer thickness of approximately 10 mm. The vacuum far − infrared parameters were: drying temperature 55°C, irradiation distance 300 mm, and vacuum level − 0.060 MPa. The ultrasound parameters were: ultrasound power 120 W and ultrasound frequencies 20/28/40 kHz.

#### 2.3.2 Different drying process experiments

Microwave vacuum drying ((US − SA) − MVD): The pretreated *Zanthoxylum bungeanum* samples, after US–SA pretreatment, were further processed by MVD equipment (Fig. 2(b)). The operating parameters were set as follows: drying temperature 55°C, vacuum level − 0.060 MPa, and rotation speed 50 Hz. To prevent localized overheating caused by nonuniform electromagnetic field distribution, a rotating tray structure was employed to enhance drying stability.

Radio frequency vacuum drying ((US − SA) − RFVD): The US − SA pretreated fresh *Zanthoxylum bungeanum* was evenly spread in a polypropylene container and then subjected to dehydration in a RFVD system (Fig. 2(c)). The specific parameters were: drying temperature 55°C, vacuum level − 0.060 MPa, and electrode spacing 80 mm.

Far–infrared vacuum drying ((US − SA) − FIRD): Fresh samples were pretreated with US–SA and subsequently dehydrated in a FIRD device. The temperature was maintained at 55°C, the irradiation height was 300 mm, and the vacuum level was − 0.060 MPa.

Multi − frequency ultrasound − far − infrared vacuum drying ((US − SA)−(MFUS − FIRD)): Fresh samples were first subjected to combined US − SA pretreatment, followed by MFUS − FIRD (Control 2).

Multi − frequency ultrasound − assisted hot air drying ((US − SA)−(MFUS − HAD)): A MFUS field was introduced concurrently with HAD drying. The operating parameters of the HAD system and the MFUS unit were: drying temperature 55°C, air velocity 1.0 m/s, ultrasound power 120 W, and ultrasound frequency 20/28/40 kHz.

### 2.4 Moisture content (*MC*) and drying rate (*DR*)

The wet basis *MC* and *DR* were calculated according to the Eq. (1), (2) [34], [35]:

(1) $$\mathrm{BI}=100\times \left[\left(a^{*}+1.75\cdot L^{*}\right)/\left(5.645\cdot L^{*}+a^{*}-3.012\cdot b^{*}\right)-0.31\right]/0.17$$

(2) $$\mathrm{DR}=\left(M_{t}-M_{t-\Delta t}\right)/\Delta t$$

where *M*_t_ denotes the *MC* on a dry basis at a given time, g/g; *M*_d_ is the absolutely dried sample, g/g; *M*_t_ and *M_t-_*_Δt_ correspond to the *MC* at time t and t-Δt, respectively, g/g.

### 2.5 Determination of energy consumption

An electric energy meter (ADL200/C, Acrel Electric Co., Ltd., Shanghai, China) was used to measure the energy consumption under different drying processes.

### 2.6 Determination of color

After pulverization, uniformly mixed powders of the *Zanthoxylum bungeanum* samples were obtained, and their *L*, *a*, and *b* values were measured using a colorimeter (CR − 400, Konica Minolta, Japan) under a D65 standard light source. Each set of samples was measured in triplicate, and the mean value was recorded. L* denotes lightness, a* reflects red–green chromaticity, b* represents yellow–blue chromaticity, and the color difference (ΔE) and browning index (BI) were used to quantify the chromatic deviation between samples dried under different conditions and fresh samples [36].

(3) $$\Delta E=\sqrt{{\left(L^{*}-L_{0}\right)}^{2}+{\left(a^{*}-a_{0}\right)}^{2}+{\left(b^{*}-b_{0}\right)}^{2}}$$

(4) $$\mathrm{BI}=\left[100\times \left(x-0.31\right)\right]/0.17$$

(5) $$C=\sqrt{{\left(a^{*}\right)}^{2}+{\left(b^{*}\right)}^{2}}$$

### 2.7 Determination of total amide content

A total of 20 g of fresh samles were homogenized, after which 5 g of the homogenate was transferred to a stoppered flask and mixed with 50 mL of methanol. The mixture underwent ultrasonic extraction for 30 min and was then cooled to ambient temperature. The extract was transferred to a 100 mL volumetric flask and brought to volume with methanol. Subsequently, 10 mL of the supernatant was transferred to a 50 mL volumetric flask for dilution. The solution was filtered through a 0.22 μm microporous membrane, and its absorbance was measured. The calculation formula is as follows:

(6) $$\mathrm{TAC}=\left(A\times K\times V\right)/\left(m\times E\right)$$

where TAC is the total amide content in the sample, mg/g; *A* is the absorbance of sample at 270 nm; *K* is the dilution factor; *V* is the final volume, mL; *m* is the sample mass, g; *E* is the absorbance of *Zanthoxylum bungeanum* at 270 nm.

### 2.8 Determination of hydroxy − α − sanshool

Hydroxy − α − sanshool was quantified using high performance liquid chromatography [37]. Chromatographic separation was performed on a C_18_ column (250 mm × 4.6 mm, 5 μm). The mobile phase consisted of acetonitrile (A) and 1% acetic acid in water (B), with gradient elution programmed as follows: 0 − 10 min, 35%−65% A; 10 − 25 min, 35%−75% A. The flow rate was 1.0 mL/min, the column temperature was maintained at 30°C, the detection wavelength was set at 270 nm, and the injection volume was 10 μL.

### 2.9 Determination of total alkaloid content

The total alkaloid content was determined using a plant alkaloid ELISA kit. According to the kit instructions, 0.5 g of sample was accurately weighed, mixed with 15 mL of phosphate buffer, ultrasonicated for 1 h, and centrifuged for 20 min. The supernatant was collected for subsequent analysis. Absorbance was recorded at 450 nm, and each sample was measured in triplicate.

### 2.10 Determination of total phenolics and total flavonoids

TPC was determined using the Folin–Ciocalteu method [38]. Dried *Zanthoxylum bungeanum* was pulverized and passed through a 60 − mesh sieve. 0.5 g of the sample was mixed with 70% ethanol, followed by ultrasonic extraction (30 min, 40 kHz, 120 W). After shaking and centrifugation, the supernatant was collected, mixed with Folin–Ciocalteu reagent, and subsequently reacted with Na_2_CO_3_ solution. The mixture was incubated at room temperature in the dark for 1 h, and absorbance was measured at 765 nm. Results were calculated using a gallic acid (GAE) standard curve.

TFC was determined using the NaNO_2_–Al(NO_3_)_3_–NaOH colorimetric method [39]. The 70% ethanol extract served as the reaction substrate, sequentially reacting with NaNO_2_, Al(NO_3_)_3_, and NaOH. Absorbance was measured at 510 nm, and results were calculated using a rutin (RE) standard curve. Each sample was analyzed in triplicate.

### 2.11 Determination of antioxidant activity

For the DPPH assay, a 0.2 mmol/L DPPH anhydrous ethanol solution was prepared [40]. A total of 3.0 mL of DPPH solution was mixed with 0.35 mL of sample solution and allowed to react in the dark for 30 min, followed by absorbance measurement at 517 nm.

For the ABTS assay, ABTS solution (7.4 mmol/L) and K_2_S_2_O_8_ solution (2.6 mmol/L) were mixed at a 1:1 vol ratio and kept in the dark at room temperature for 12 h to generate the ABTS stock solution. The stock solution was diluted with anhydrous ethanol to obtain a working solution with an absorbance of 0.70 ± 0.02 at 734 nm. For measurement, 800 μL of ABTS working solution was mixed with 40 μL of sample solution, reacted in the dark for 30 min at room temperature, and the absorbance was recorded at 734 nm [41].

For the FRAP assay, the FRAP working solution was prepared by mixing 300 mmol/L acetate buffer (pH 3.6), 10 mmol/L TPTZ solution, and 20 mmol/L FeCl_3_·6H_2_O solution at a 10:1:1 vol ratio. A total of 1.0 mL of the working solution was mixed with 70 μL of sample solution, reacted in the dark for 30 min at room temperature, and the absorbance was measured at 593 nm [42]. All experiments included blank controls and were performed in triplicate.

### 2.12 Gas chromatography–mass spectrometry (GC–MS) analysis

Volatile constituents in *Zanthoxylum bungeanum* were analyzed using HS − SPME − GC − MS [8], [43]. A total of 1.00 g of powdered pericarps was placed in a 20 mL headspace vial and sealed, followed by SPME pretreatment. SPME conditions were as follows: the system was maintained at 65°C, equilibrated for 20 min, and extracted for 30 min; the fiber was then thermally desorbed in the injection port for 3 min. Chromatographic conditions were: injection port temperature 250°C; capillary column HP − 5MS (30 m × 0.25 mm × 0.25 μm); carrier gas helium at 1.0 mL·min^g−1^. The oven program was: initial 40°C held for 2 min, increased to 50°C at 0.8°C·min^g−1^ and held for 5 min, then increased to 200°C at 3.5°C·min^g−1^ and maintained until completion. Mass spectrometric parameters were: EI ionization source, ion source temperature 230°C, interface temperature 250°C, full − scan mode with *m*/*z* range 35 − 500 amu, electron impact energy 70 eV, and solvent delay 3.0 min.

### 2.13 Electronic nose analysis

For each drying treatment, 5 g of *Zanthoxylum bungeanum* were accurately weighed, placed in a 50 mL headspace vial, sealed, and equilibrated for 30 min to allow volatile compounds to reach headspace equilibrium before analysis. Electronic nose profiling was performed using a PEN 3 portable system (Air Sense Analytics Co., Schwerin, Germany) equipped with ten metal oxide semiconductor sensors, each exhibiting characteristic responses to specific classes of volatile compounds: W1C to aromatic hydrocarbons; W5S to nitrogen oxides; W3C to ammonia and amines; W6S to long chain hydrocarbons; W5C to alkanes and aromatic compounds with a broad sensitivity range; W1S to short chain hydrocarbons; W1W to sulfur − containing compounds and terpenes; W2S to alcohols and certain aldehydes and ketones; W2W to aromatic and sulfur − containing compounds; and W3S primarily to long chain alkanes. Signals collected at 110 s were used for analysis. Operating parameters were: flushing time 180 s, zeroing time 5 s, sample preparation time 5 s, and injection time 120 s. The carrier gas was dry air at a flow rate of 300 mL/min [3], [44]. Five parallel samples were prepared for each treatment, and each sample was analyzed in triplicate.

### 2.14 Determination of microstructural

To evaluate the effects of different drying methods on the microstructure of *Zanthoxylum bungeanum*, dried samples were cut into pieces of approximately 3 mm × 3 mm, mounted on stubs, sputter − coated with metal, and examined using scanning electron microscopy (SEM). Observations were conducted at an accelerating voltage of 5 kV, and micrographs were acquired at 500 × magnification to document surface morphology and cell wall features, enabling comparison of microstructural differences among drying treatments [45].

### 2.15 Sensory evaluation

Sensory evaluation was performed using a 10 point scoring system by a trained panel of 20 assessors (10 males and 10 females) in a standardized sensory laboratory maintained at 25°C and free of extraneous odors. A blind assessment was conducted. Based on the sensory attributes characteristic of *Zanthoxylum bungeanum*, the evaluated parameters included taste attributes (numbing sensation, spiciness, bitterness, and off − odor), aroma characteristics (fresh aroma, pungent aroma, and citrusy aroma), color, appearance, and overall acceptance. Each attribute was scored on a scale from 1 to 10, where 1 indicated extreme dislike and 10 indicated extreme liking. The mean value across assessors was used as the final score for each parameter.

## 3 Results and discussion

### 3.1 Drying characteristics

**Fig. 3:**
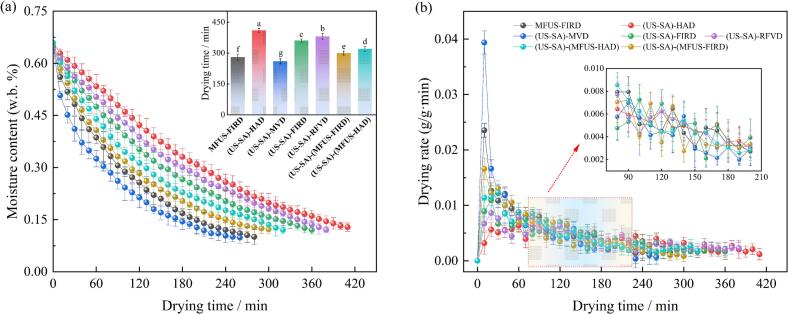
*MR* (a) and *DR* (b) of *Zanthoxylum bungeanum* under different drying treatments.

Drying behavior is a key indicator for characterizing heat and mass transfer performance in coupled energy field drying systems, reflecting moisture migration rates, heat and mass transfer efficiency, and structural evolution. The drying curves of *Zanthoxylum bungeanum* under different drying methods exhibited typical falling rate patterns. However, both drying rate and total drying time differed significantly (*P* < 0.05). The drying durations of (US − SA)−(MFUS − FIRD) and (US − SA)−(MFUS − HAD) were 300 min and 320 min, representing reductions of 16.70% and 26.8% relative to (US − SA) − FIRD (360 min) and (US − SA) − HAD (410 min), respectively (Fig. 3), indicating that the coupled energy fields markedly enhanced energy transfer and moisture diffusion. This enhancement effect is not only attributed to the reduction of boundary-layer resistance induced by ultrasound, but is also closely associated with changes in the internal moisture migration pattern under multi-frequency acoustic fields. Zang et al. [22] found that multi-frequency ultrasound could generate a more uniform acoustic energy distribution through frequency superposition, thereby reducing local standing-wave and energy accumulation phenomena under single-frequency ultrasound and improving the internal energy utilization efficiency of *Cornus officinalis* tissues. The superposition of multi-frequency waves generates a broadband energy distribution and establishes interlaced pressure fields at heterogeneous interfaces, thereby inducing cyclic compression–relaxation of localized microdomains, alleviating standing-wave–induced energy attenuation, and ultimately enhancing mass transfer efficiency [46]. This process also promotes microcracking of cell walls and cuticular layers, reducing diffusion resistance and facilitating moisture migration through capillary channels and intercellular spaces. Concurrently, cavitation induced transient high pressure and microjets cause localized tissue loosening, substantially accelerating moisture diffusion and shortening drying time [47]. Among all treatments, (US − SA) − MVD exhibited the shortest drying time (260 min), a 36.6% reduction compared with (US − SA) − HAD. This is attributable to the volumetric heating mechanism of MVD, in which electromagnetic waves directly interact with polar water molecules, inducing rapid dipole rotation and internal heat generation. Because energy propagates outward from the interior, the vaporization front forms within the sample, resulting in a high initial drying rate. Zeng et al. [26] similarly demonstrated that MVD could significantly improve drying efficiency and product quality during the MVD of ginger. However, the highly concentrated energy input can produce localized hotspots, leading to puffing or surface overdrying, which subsequently reduces drying efficiency, causes moisture entrapment, and may induce local rewetting [48]. Compared with MFUS − FIRD (280 min), the drying time of (US − SA)−(MFUS − FIRD) increased by approximately 7.1%, primarily due to the mass transfer modulation induced by the SA coating in the early drying stage. Under ultrasonic assistance, SA formed a uniform and dense semipermeable film on the surface of *Zanthoxylum bungeanum*, restricting rapid evaporation of free surface water at the initial stage and increasing external diffusion resistance at the gas–solid interface, thereby reducing early evaporation flux. The high hydrophilicity of the SA layer enhances water − vapor transport resistance after swelling, delaying the establishment of surface moisture gradients and slowing early stage moisture migration. Although it manifested macroscopically as a slight prolongation of drying time, it effectively prevented the development of surface hardening and the “case-hardening effect”, improved the coordination between internal and external diffusion, and provided a more stable mass-transfer pathway for sustained dehydration during the middle and later stages. Similar findings were also reported by Santanu et al. [49].

### 3.2 Energy consumption analysis

**Fig. 4:**
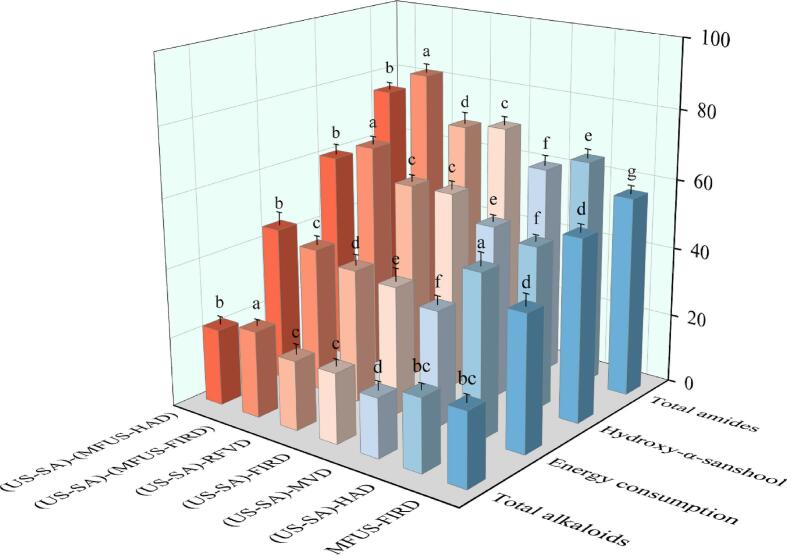
Energy consumption, total amides, hydroxy-α-sanshool, and total alkaloid contents of *Zanthoxylum bungeanum* after US–SA pretreatment combined with different drying processes.

Energy consumption is not only a key indicator for evaluating the performance of agricultural product drying processes but also an essential metric reflecting process economics, environmental compatibility, and technological advancement [50]. Efficient drying systems substantially reduce energy consumption while improving throughput. The results showed that the specific energy consumption of all treatments ranged from 33.25 to 46.07 kW·h·kg^g−1^, exhibiting significant process dependent differences (*P* < 0.05). The ranking was: (US − SA) − HAD > (US − SA)−(MFUS − HAD) > (US − SA)−(MFUS − FIRD) > (US − SA) − RFVD > MFUS − FIRD > (US − SA) − FIRD > (US − SA) − MVD (Fig. 4). Conventional hot air drying exhibited the highest energy consumption due to its single heat transfer mechanism, large thermal capacity of the air medium, prolonged drying duration, and low convective heat − transfer efficiency, with substantial energy loss occurring during air circulation and surface evaporation. In contrast, FIRD, RFVD, and MVD based on radiative or volumetric heating − significantly improved heat transfer pathways. MVD showed the lowest energy consumption (33.25 kW·h·kg^g−1^), 27.83% lower than (US − SA) − HAD, owing to its volumetric heating mechanism in which electromagnetic energy directly interacts with internal water molecules, enhancing drying efficiency and reducing convective losses. However, non − uniform energy distribution, localized overheating, and tissue collapse restrict its overall energy utilization and product quality stability. The energy consumption of (US − SA) − FIRD was 36.77 kW·h·kg^g−1^, 20.19% lower than that of HAD. Far-infrared radiation can be efficiently absorbed by water molecules and rapidly converted into thermal energy, thereby enhancing surface heating efficiency. Nevertheless, owing to the limited penetration depth of far-infrared radiation, internal moisture migration remains restricted by temperature gradients and diffusion resistance. Xu et al. [50] reported similar findings in drying experiments on Anoectochilus. Incorporating MFUS further homogenized the energy field. The energy consumption of (US − SA)−(MFUS − FIRD) reached 40.57 kW·h·kg^g−1^, 10.33% higher than (US − SA) − FIRD, yet accompanied by a 16.67% reduction in drying time. This indicates that multi − frequency acoustic fields enhanced moisture migration and heat–mass coupling efficiency, but also introduced additional energetic demand. This increase likely reflects the need for supplemental acoustic power and the intrinsic attenuation of sound energy in the medium, where part of the acoustic energy is dissipated as ineffective heat due to acoustic impedance loss. Notably, compared with (US − SA) − HAD, (US − SA)−(MFUS − HAD) reduced energy consumption by 5.19%. The cavitation and microstreaming generated by MFUS weakened the external boundary layer resistance and enhanced internal diffusion, allowing heat flux to be more effectively directed into the sample while reducing heat accumulation and localized energy dissipation. Consequently, moisture removal per unit time increased, yielding improved dehydration efficiency and lower energy use. The results also revealed that MFUS − FIRD consumed less energy than (US − SA)−(MFUS − FIRD). This difference is attributed to the semi − permeable SA layer formed during US − SA pretreatment, which functioned as a hydrated polymer network that restricted surface moisture evaporation and increased external mass transfer resistance during the early drying stage. This membrane induced resistance elevated local thermal resistance and impeded heat transfer, thereby reducing early stage thermal efficiency. Although this phenomenon manifested as increased energy consumption at the macroscopic scale, it simultaneously prevented case hardening and protected heat sensitive constituents, providing notable quality advantages.

Furthermore, from the perspective of industrial application, the US − SA composite pretreatment combined with MFUS − VFIR drying demonstrated considerable application potential and engineering feasibility for the high-quality processing of *Zanthoxylum bungeanum*. On the one hand, the coupling of MFUS and VFIR could significantly enhance heat and mass transfer efficiency, shorten the drying time, and improve energy utilization efficiency, thereby alleviating the high energy consumption and prolonged processing time commonly associated with conventional HAD. On the other hand, SA possesses excellent biosafety, biodegradability, and cost-effectiveness, making it highly suitable for large-scale industrial implementation. Therefore, the proposed composite drying strategy exhibits promising potential for process scale-up and industrial application.

### 3.3 Color analysis

**Fig. 5:**
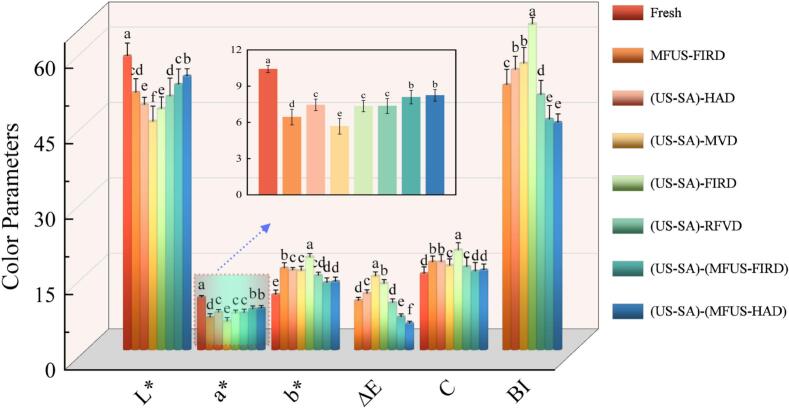
Effects of US–SA pretreatment combined with different drying processes on the color of *Zanthoxylum bungeanum*.

Color is a key sensory attribute for evaluating the drying quality of *Zanthoxylum bungeanum*, as it directly reflects visual appeal and pigment retention. Fresh samples exhibited L*, a*, and b* values of 58.45, 10.42, and 11.02, corresponding to their characteristic reddish − brown hue (Fig. 5). After drying, L* values ranged from 45.42 to 54.44. The (US − SA)−(MFUS − HAD) and (US − SA)−(MFUS − FIRD) samples showed the highest brightness, whereas (US − SA) − MVD exhibited the most pronounced discoloration. This may be attributed to the cavitation, microjets, and multi–frequency superimposed acoustic pressure generated during MFUS, which improve heat and mass transfer uniformity, suppress local overheating, and inhibit thermal degradation of anthocyanins and carotenoids, thereby enhancing brightness. In contrast, the concentrated energy density and nonuniform dielectric heating in MVD readily generate hotspots, leading to surface charring and browning accumulation. Compared with (US − SA) − MVD, L* values of (US − SA) − RFVD and (US − SA) − HAD increased by 7.38% and 11.05%, indicating that RFVD and HAD moderated browning reactions and better retained brightness. Xu et al. [51] similarly reported in a study on RFVD − HAD combined drying of wolfberry that both RFVD and HAD effectively preserved sample color while mitigating browning and oxidative deterioration. The a* and b* parameters reflect pigment stability and chromatic shifts of *Zanthoxylum bungeanum*. The (US − SA)−(MFUS − HAD) samples showed the highest a* value (8.24), followed by (US − SA)−(MFUS − FIRD), suggesting that cavitation and microstreaming under MFUS improved heat and mass transfer coupling, reduced surface temperature gradients during hot air and far − infrared heating, and slowed oxidative pigment degradation. The b* values increased overall, indicating enhanced yellowness after drying. The (US − SA) − FIRD samples showed the highest b* value (18.44), followed by (US − SA) − MVD (15.77), reflecting marked yellowing. This primarily results from heat − induced carotenoid oxidation and Maillard reaction product accumulation; the former produces β–charge transfer type oxidation products, and the latter forms pyrazines and furfural derivatives with yellow–brown hues, jointly increasing b*.

The ΔE values of (US − SA)−(MFUS − FIRD) and (US − SA)−(MFUS − HAD) were 41.48% and 53.26% lower than those of (US − SA) − HAD, demonstrating that MFUS effectively suppressed nonenzymatic browning, phenolic oxidation, and anthocyanin degradation. Multi–frequency acoustic fields facilitate cyclic pressure − driven renewal at gas–liquid interfaces and promote uniform moisture migration, reducing surface heat accumulation. Cavitation further limits polyphenol oxidase activity and weakens free − radical chain reactions, thereby reducing color difference development [52]. Relative to MFUS − FIRD, ΔE and BI of (US − SA)−(MFUS − FIRD) decreased by 33.06% and 13.00%, respectively. This may arise from the uniform edible film formed during US − SA pretreatment; its hydrogen bonded network of carboxyl and hydroxyl groups stabilizes the conjugated structure of anthocyanins, delaying deglycosylation and ring opening reactions and thus inhibiting both enzymatic and nonenzymatic browning. Concurrently, ultrasonic cavitation enhanced mass transfer between the intracellular and extracellular compartments, increased the rate of moisture migration, and shortened the duration of sample exposure to high-temperature conditions, thereby reducing the degradation of anthocyanins and other thermolabile pigments and ultimately preserving superior color quality. Fatemeh et al. [53] also reported that coating application coupled with the drying process significantly improved the color parameters of the kiwifruit.

### 3.4 Total amide content analysis

Total amides constitute the primary chemical basis for the characteristic pungent tingling flavor of *Zanthoxylum bungeanum*, and their abundance directly determines the sensory intensity and bioactivity of dried *Zanthoxylum bungeanum*. As shown in Fig. 4, different drying methods produced significant variations in total amide retention (*P* < 0.05), with values ranging from 57.48 ± 3.67 to 82.32 ± 5.64 mg/g, indicating that energy − transfer modes and oxidative environments are decisive factors governing the structural stability of pungent amides in *Zanthoxylum bungeanum*. The total amide content of the (US − SA)−(MFUS − HAD) sample was 75.43 ± 4.37 mg/g, representing a 15.46% increase relative to (US − SA) − HAD (65.33 ± 4.61 mg/g). This indicates that incorporating MFUS into HAD improves heat − transfer uniformity and suppresses oxidative reactions. HAD relies on strong convective heating, which induces rapid surface temperature rise and high oxygen partial pressure. Under such conditions, the electrophilicity of the carbonyl carbon in amide structures increases, facilitating nucleophilic addition or radical − initiated reactions with oxygen. These reactions generate amide radicals that subsequently undergo β − scission or deamination, producing amines or imine derivatives and leading to decreased total amide content. MFUS homogenizes the energy field and enhances internal heat conduction, thereby reducing localized thermal stress and oxidative load and slowing amide thermolysis. Notably, MFUS − FIRD resulted in the lowest total amide content (57.48 ± 3.94 mg/g), 30.17% lower than (US − SA)−(MFUS − FIRD), demonstrating the significant advantage of ultrasound − assisted SA coating in maintaining amide stability. SA formed a dense yet moderately permeable biopolymer film on the sample surface, maintaining a relatively humid micro − environment during the early drying stage. Nida et al. [54] likewise demonstrated the superior effectiveness of edible coatings in preserving product quality. This moderated the surface temperature rise and reduced thermally induced amide bond cleavage and alkyl chain scission/rearrangement. Moreover, hydrogen bonding between the carboxyl groups of SA and the phenolic hydroxyl or amino groups on the *Zanthoxylum bungeanum* surface generated transient intermolecular interactions during heating, providing further protection to the amide moieties. Compared with (US − SA) − FIRD (70.56 ± 5.02 mg/g), the total amide content of (US − SA)−(MFUS − FIRD) increased by 16.67%. This enhancement is attributed to the composite energy field generated by MFUS within the FIRD system, which modulates the resonance frequencies of polar regions at the molecular level, thereby maintaining amide bonds under reduced local tensile stress and lowering the probability of bond rupture. Concurrently, ultrasound − induced pressure oscillations and microscale stress relaxation stabilize the electron density around organic amine groups, preventing carbonyl activation, ring opening, or isomerization of the amide structure.

### 3.5 Hydroxy − α − sanshool analysis

Amide compounds are the principal contributors to the numbing sensation of *Zanthoxylum bungeanum*, most of which are linear unsaturated fatty acid amides; among them, hydroxy − α − sanshool is representative, exhibiting strong pungency and serving as a key quality indicator. Across the different drying treatments, hydroxy − α − sanshool contents ranged from 47.37 ± 3.12 to 65.59 ± 4.68 mg/g (Fig. 4). The highest content was detected in samples subjected to (US − SA)−(MFUS − FIRD) (65.59 ± 4.68 mg/g), representing a 24.58% increase relative to MFUS − FIRD (52.65 ± 3.44 mg/g), indicating that US − SA pretreatment markedly enhanced the structural stability of hydroxy − α − sanshool. This stabilization resulted from the SA coating moderately reducing outward moisture diffusion during early drying, generating a more uniform internal temperature rise and preventing oil glands and lipid microdomains from experiencing transient high − temperature and oxidative exposure. Consequently, thermo oxidative scission and structural rearrangement of the unsaturated amide chain in hydroxy − α − sanshool were minimized, thereby improving its retention. Simultaneously, the progressive microstructural relaxation induced by MFUS promoted more gradual depolymerization of trichomes and adjacent oil gland tissues, avoiding abrupt local rupture and enabling a smoother transition of amides from bound to releasable states, thus reducing hydroxy − α − sanshool loss caused by sudden tissue breakage and oxidative escape. Hydroxy − α − sanshool contents in (US − SA) − FIRD and (US − SA) − RFVD samples were 57.05 ± 2.12 and 56.86 ± 4.09 mg/g, respectively, with no significant difference (*P* > 0.05). Although both values were substantially higher than those in (US − SA) − HAD, they remained considerably lower than in (US − SA)−(MFUS − FIRD). This indicates that vacuum conditions mitigate oxidation but, without the controllable micro − disruption provided by MFUS, effective amide release remains limited. Hydroxy − α − sanshool in (US − SA)−(MFUS − HAD) reached 60.30 ± 4.30 mg/g, a 27.30% increase over (US − SA) − HAD, demonstrating that MFUS markedly delayed the thermal cleavage and isomerization of hydroxy − α − sanshool, thereby enhancing amide retention. Notably, hydroxy − α − sanshool content in (US − SA) − MVD was 11.64% lower than in (US − SA) − RFVD, attributable to the non − uniform energy deposition of the microwave field in high moisture tissues, which imposed transient stresses on glandular structures and accelerated volatilization and thermally induced degradation of key amide compounds, resulting in markedly reduced retention.

### 3.6 Total alkaloid content analysis

The total alkaloid content reflects the impact of different drying methods on the stability of bioactive constituents in *Zanthoxylum bungeanum*. In MFUS − FIRD samples, alkaloid content was 21.27 ± 0.89 ng/g, whereas after (US − SA)−(MFUS − FIRD) treatment it increased to 24.89 ± 0.36 ng/g, representing a 17.02% elevation (Fig. 4). This indicates that the semipermeable hydrogel film formed by the US − SA pretreatment altered the interfacial mass transfer conditions during the early drying stage, reducing surface evaporation flux and establishing a milder temperature gradient, thereby mitigating alkaloid oxidative cleavage in the high moisture phase. Meanwhile, the weakly polar network formed by the carboxyl groups and chain segments of SA may provide transient micro − environmental buffering around trichomes, shielding hydroxyl − substituted pyridine ring structures from direct cavitation microjet impact and enhancing their structural stability. In contrast, alkaloid contents in (US − SA) − FIRD and (US − SA) − RFVD samples decreased to 20.18 ± 0.62 and 19.98 ± 0.43 ng/g, respectively, with no significant difference (*P* > 0.05). The lowest alkaloid content was observed in (US − SA) − MVD samples (17.07 ± 0.55 ng/g), representing an 18.60% reduction compared with (US − SA) − HAD. This suggests that hotspots generated by electromagnetic energy in water enriched regions substantially raise local activation energy, promoting chain scission or deamination reactions involving amide bonds and pyridine ring structures, thereby reducing alkaloid content. Notably, coupling MFUS with different drying techniques exerted a positive effect on alkaloid retention in *Zanthoxylum bungeanum*. For example, alkaloid contents in (US − SA)−(MFUS − HAD) and (US − SA)−(MFUS − FIRD) samples reached 22.01 ± 0.37 ng/g and 24.89 ± 0.36 ng/g, respectively, representing increases of 4.96% and 18.69% over (US − SA) − HAD. This indicates that the multi–frequency acoustic field enhanced intercellular gap expansion and uniform thermal diffusion, effectively reducing oxidative accumulation of alkaloids during the later drying stage.

### 3.7 Total phenolic and total flavonoid contents

**Fig. 6:**
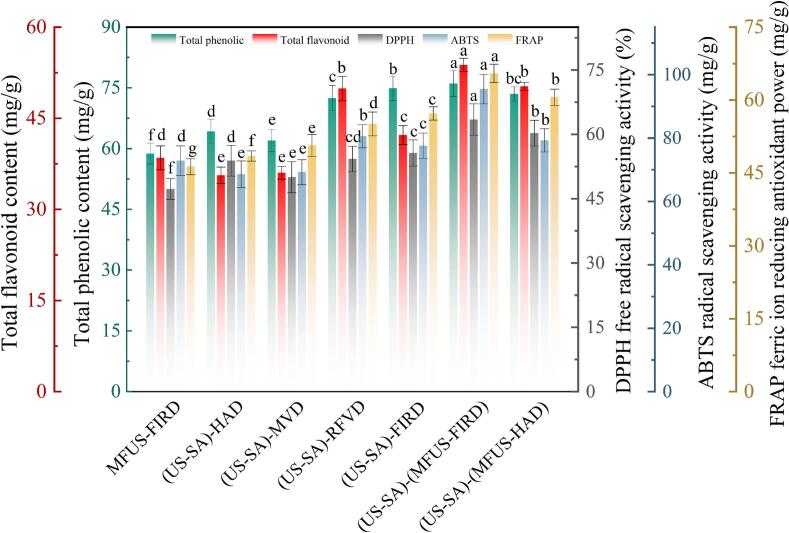
Changes in total phenols, total flavonoids, and antioxidant capacity of *Zanthoxylum bungeanum* after US–SA pretreatment combined with different drying processes.

Phenolic and flavonoid compounds are the major antioxidant constituents in *Zanthoxylum bungeanum*, exerting critical influence on its color, flavor, nutritional quality, and storage stability. After different drying treatments, total phenolic contents ranged from 58.77 ± 4.97 to 76.07 ± 5.65 mg/g, and total flavonoid contents ranged from 35.41 ± 1.64 to 53.78 ± 3.02 mg/g (Fig. 6). In (US − SA)−(MFUS − FIRD) dried samples, total phenolics and total flavonoids reached 76.07 ± 5.65 and 53.78 ± 3.02 mg/g, respectively, representing increases of 29.45% and 39.73% relative to MFUS − FIRD. This indicates that (US − SA) pretreatment conferred a pronounced advantage in preserving bioactive constituents. Ultrasonic treatment disrupted the cuticular layer through cavitation and microjets, facilitating the conversion of phenolic and flavonoid precursors from bound to free forms and enhancing their release during drying. Concurrently, acoustic agitation accelerated internal moisture diffusion and improved thermal uniformity, thereby reducing oxidative losses caused by localized thermal accumulation. Furthermore, the SA coating formed a stable microenvironment during the early drying stage by lowering oxygen permeability and modulating surface heat transfer rates, weakening oxidative chain reactions and enhancing the structural stability and thermo − resistance of phenolics and flavonoids [55]. In (US − SA)−(MFUS − HAD) samples, total phenolic and flavonoid contents were 73.48 and 50.24 ± 2.69 mg/g, corresponding to increases of 14.36% and 41.84% compared with (US − SA) − HAD. The synergistic multi–frequency acoustic field alleviated the limitations of uneven energy distribution inherent in conventional drying, generating persistent microflow perturbations and cavitation, which enhanced coupled heat–mass transfer and reduced local heat buildup and oxidative stress. Transient high pressures and microscale shear forces generated by acoustic cavitation increased molecular diffusion rates, contributing to reduced polyphenol oxidase (PPO) activity and suppression of phenolic oxidation chains, ultimately slowing oxidative degradation of bioactive constituents. Wang et al. [56] likewise found, in their study on *Astragalus* mead, that the application of ultrasound could significantly enhance the retention of total phenolics. Compared with (US − SA) − HAD, total phenolic and flavonoid contents in (US − SA)−(MFUS − FIRD) and (US − SA) − RFVD samples increased by 18.43% and 51.83%, and 16.57% and 19.27%, respectively. High temperature and oxygen rich conditions during HAD accelerated enzymatic and thermally induced oxidation of phenolics, while quinone intermediates formed during oxidation further polymerized and crosslinked, leading to structural degradation and diminished antioxidant capacity. Lin et al. [57] also reported similar findings in a study on orange peel drying, demonstrating that hot air drying (HAD) exerted the greatest impact on enzymatic activity, thereby leading to reductions in total phenolic and total flavonoid contents. By contrast, MFUS − FIRD and RFVD significantly improved heat–mass transfer uniformity and effectively suppressed radical chain reactions under low − oxygen conditions, resulting in markedly higher retention of phenolics and flavonoids. Notably, total phenolic and total flavonoid contents in (US − SA) − MVD dried samples were relatively low, at 62.01 ± 4.22 mg/g and 36.04 ± 1.37 mg/g, respectively. This was attributed to strong interactions between microwaves and polar molecules, which caused rapid vibration of free water within cells and generated intense localized heating, leading to structural cleavage of some bioactive components. Additionally, non − uniform energy distribution created localized hotspots, inducing phenolic polymerization and thermal degradation of flavonoids.

### 3.8 Antioxidant capacity analysis

Antioxidant capacity is a key indicator of the preservation of bioactive components in *Zanthoxylum bungeanum*. Different drying processes produced significant differences in antioxidant capacity (*P* < 0.05). After MFUS − FIRD and (US − SA)−(MFUS − FIRD), the DPPH, ABTS, and FRAP values of *Zanthoxylum bungeanum* were 47.24%, 72.87 mg Trolox/g, 46.28 mg Fe^2+^/g and 63.44%, 95.47 mg Trolox/g, 65.48 mg Fe^2+^/g (Fig. 6), respectively, indicating that (US − SA) pretreatment enhanced the stability of the antioxidant system. Ultrasonic cavitation and mechanical effects disrupted the waxy cuticular layer of *Zanthoxylum bungeanum*, facilitating the release of phenolic and flavonoid compounds; simultaneously, the semipermeable layer formed by SA restricted oxygen diffusion during the early drying stage, mitigating oxidative stress and jointly suppressing PPO activity, thereby reducing the conversion of phenolic hydroxyl groups to quinones and improving antioxidant capacity. Relative to (US − SA) − FIRD, the DPPH, ABTS, and FRAP values of (US − SA)−(MFUS − FIRD) increased by 14.50%, 15.63%, and 17.56%, while total phenolic and total flavonoid contents increased by 5.23% and 7.81%. This is attributable to the broadband energy distribution generated by MFUS, which formed intersecting interference fields between the drying medium and sample tissue, promoting uniform energy transfer and synchronized molecular vibration. These effects reduced localized overheating and the propagation of radical chain reactions, thereby delaying the auto − oxidation of phenolic and flavonoid compounds [58]. Meanwhile, cavitation and microstreaming induced by the multi–frequency acoustic field disrupted microstructural elements of the cell wall and improved heat–mass transfer uniformity, decreasing phenolic hydroxyl cleavage and flavonoid isomerization in high temperature zones, ultimately enhancing antioxidant capacity. Furthermore, microscale mechanical stress and shear waves generated within the tissue by the multi–frequency acoustic field weakened PPO activity and facilitated the redistribution of reductive constituents (e.g., polyphenolic hydroxyl groups) among cells, enabling a broader involvement of electron donors in radical scavenging reactions, thereby increasing the responses of DPPH and FRAP [59]. The (US − SA) − MVD group exhibited comparatively low antioxidant activity (DPPH = 50.01%, ABTS = 69.25 mg Trolox/g, FRAP = 50.65 mg Fe^2+^/g), only slightly higher than HAD. Although the vacuum environment partially suppressed oxidative reactions, the pronounced non − uniformity of microwave energy resulted in localized overheating, leading to phenolic hydroxyl cleavage, flavonoid isomerization, and polymerization, and thus a marked reduction in antioxidant capacity. This aligns with the lower total phenolic and flavonoid contents, indicating that dielectric heating non − uniformity is the principal factor driving antioxidant loss. In (US − SA) − HAD *Zanthoxylum bungeanum*, the DPPH, ABTS, and FRAP values were 11.96%, 15.62%, and 12.50% lower than those of (US − SA)−(MFUS − HAD). The prolonged dehydration in (US − SA) − HAD allowed enzymatic and non − enzymatic oxidation to proceed concurrently, and the resultant quinones readily polymerized or crosslinked with proteins and carbohydrates, leading to a substantial decline in antioxidant activity [60].

### 3.9 Volatile compound analysis

**Fig. 7:**
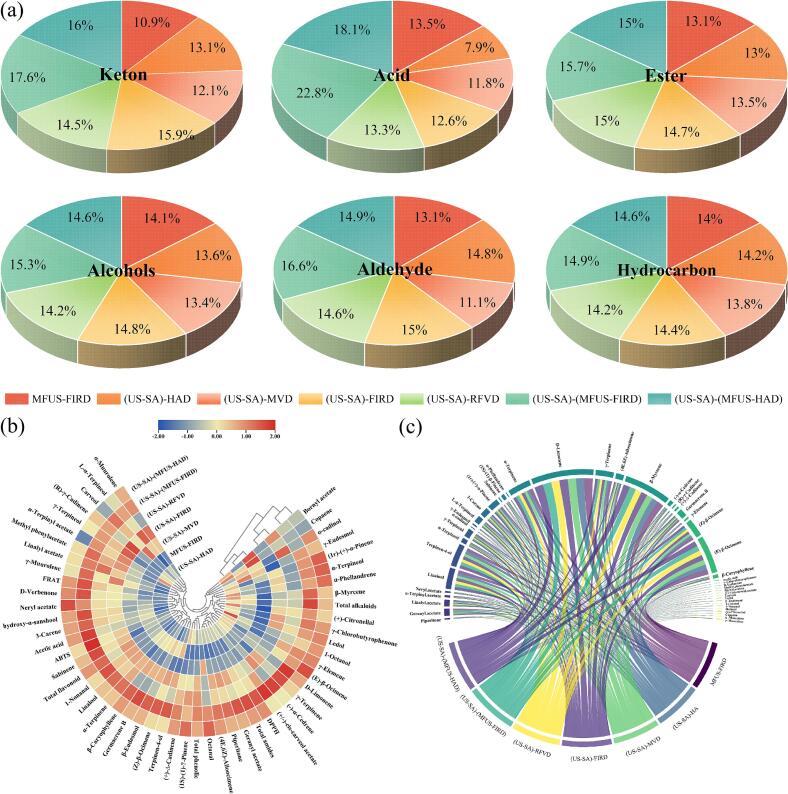
Proportions of major constituent categories (a), correlation heatmap (b), and chord diagram analysis (c) of *Zanthoxylum bungeanum* subjected to US-SA pretreatment combined with different drying techniques..

Volatile compounds constitute the primary chemical basis underlying the characteristic aroma and sensory attributes of *Zanthoxylum bungeanum*, and their composition and relative abundance are affected by drying processes and energy − field conditions. GC–MS analysis identified 82 ∼ 88 volatile compounds across the dried samples, with 46 shared key volatiles among all treatments (Table 1 and Fig. 7(a)). Alkenes showed the highest abundance, followed by alcohols, esters, aldehydes, and acids, indicating that alkenes and alcohols are the dominant volatile constituents in dried *Zanthoxylum bungeanum*, consistent with the findings of Wu et al [61]. Linalool, terpinen − 4 − ol, γ − terpineol, α − terpinene, D − limonene, γ − terpinene, β − myrcene, (E) − β − ocimene, and (Z) − β − ocimene − identified as the major volatiles in *Zanthoxylum bungeanum* − exhibited their highest relative abundance in (US − SA)−(MFUS − FIRD), with increases over MFUS − FIRD of 8.80%, 5.05%, 5.99%, 4.42%, 3.58%, 7.22%, 1.79%, 4.45%, and 5.53%, respectively. This is attributable to the cavitation and microstreaming induced by MFUS, which promoted mechanical rupture of oil glands and glandular trichomes, enhancing the effective transport of both high molecular weight and low molecular weight volatiles from the intercellular matrix to the gas–solid interface, thereby increasing the release of sesquiterpenes and related constituents. Meanwhile, the semipermeable hydrogel network formed by US − SA pretreatment in the early drying stage increased surface diffusion resistance, thus slowing the instantaneous large scale evaporation of monoterpenes particularly highly volatile monoterpene hydrocarbons and reducing their oxidative cleavage under high temperature, oxygen − rich conditions, leading to improved retention of oxygenated terpenols and esters. Compared with (US − SA)−(MFUS − FIRD), the linalool and terpinen − 4 − ol contents in (US − SA) − HAD were 387.92 ± 12.07 μg/g and 443.71 ± 10.45 μg/g, respectively, representing decreases of 16.98% and 11.04%. This indicates that dehydrogenation, isomerization, and oxidative cleavage reactions occurring under high − temperature, oxygen − rich conditions markedly reduced the fresh aroma profile of *Zanthoxylum bungeanum*, consistent with reports that HAD accelerates terpenol oxidation [62]. Compared with (US − SA) − FIRD and (US − SA) − RFVD, the retention of volatile compounds after (US − SA)−(MFUS − HAD) was higher, suggesting that MFUS facilitated more rapid and stable convective heat transfer and accelerated internal moisture migration, enabling faster transport of volatiles from highly reactive regions to the evaporation interface, thereby reducing their residence time in oxidation − prone micro − environments. Moreover, microstreaming enhanced cavitation generated by MFUS increased cell wall porosity, accelerating the conversion of terpenes from bound to free states and further improving the extractability and retention of key aroma constituents. Notably, after (US − SA) − MVD, *Zanthoxylum bungeanum* showed the lowest contents of (Z) − β − ocimene, germacrene B, γ − terpinene, and terpinen − 4 − ol, which were 345.97 ± 4.28, 143.97 ± 4.97, 352.77 ± 3.89, and 429.37 ± 9.61 μg/g, respectively. This may be attributed to the transient high energy density hotspots formed in regions of high dielectric loss during MVD, causing instantaneous temperature rises that triggered the cleavage, oxidation, or volatilization loss of heat − sensitive monoterpene hydrocarbons, oxygenated terpenols, and esters. Compared with radiative or convective heating, the internal temperature gradient generated by microwaves is considerably steeper, imposing rapid expansion rupture mechanical stress on oil gland cells and glandular trichomes, which leads to instantaneous release of encapsulated volatiles and their large scale escape driven by elevated internal vapor pressure. In addition, the heterogeneous distribution of microwave induced electromagnetic energy may accelerate lipid peroxidation cascades, converting certain sesquiterpenes and esters into aldehydes, ketones, or low − molecular − weight acids, thereby markedly compressing the overall volatile profile. Similar conclusions were likewise reported by Carla et al. [63].

**Table 1:** Relative contents (μg/g) of volatile compounds in *Zanthoxylum bungeanum* after US–SA pretreatment combined with different drying processes.

No.	Compound	Drying methods and Relative content (μg/g)						
		MFUS-FIRD	(US-SA)-HAD	(US-SA)-MVD	(US-SA)-FIRD	(US-SA)-RFVD	(US-SA)-(MFUS-FIRD)	(US-SA)-(MFUS-HAD)
Acid								
1	Acetic acid	7.45 ± 0.45^c^	4.35 ± 0.36^e^	6.49 ± 0.62^d^	6.97 ± 0.21^cd^	7.32 ± 0.37^c^	12.58 ± 0.22^a^	10.01 ± 0.59^b^
Keton								
2	γ-Chlorobutyrophenone	1.48 ± 0.06^f^	1.95 ± 0.04^d^	1.06 ± 0.07^g^	2.26 ± 0.03^c^	1.74 ± 0.03^e^	2.47 ± 0.10^a^	2.31 ± 0.13^b^
3	D-Verbenone	3.24 ± 0.14^e^	3.05 ± 0.23^g^	4.12 ± 0.09^d^	4.38 ± 0.10^c^	4.29 ± 0.18^c^	5.97 ± 0.25^a^	5.38 ± 0.16^b^
4	Piperitone	18.79 ± 2.03^e^	23.15 ± 1.94^c^	20.94 ± 1.08^d^	27.63 ± 3.40^b^	25.12 ± 2.01^bc^	29.40 ± 1.94^a^	26.76 ± 2.15^b^
Ester								
5	Methyl phenylacetate	1.34 ± 0.01^e^	1.35 ± 0.05^e^	1.79 ± 0.01^d^	2.44 ± 0.07^b^	2.31 ± 0.04^bc^	2.67 ± 0.01^a^	2.07 ± 0.03^c^
6	Geranyl acetate	138.48 ± 4.32^d^	142.68 ± 3.94^c^	139.67 ± 6.42^d^	147.28 ± 2.49^b^	143.55 ± 3.88^c^	151.37 ± 5.27^a^	147.01 ± 4.32^b^
7	Linalyl acetate	124.37 ± 3.20^e^	115.08 ± 3.67^f^	129.44 ± 4.31^d^	139.57 ± 2.54^b^	151.27 ± 4.92^a^	149.57 ± 3.22^a^	136.88 ± 4.67^c^
8	Bornyl acetate	3.37 ± 0.10^e^	4.67 ± 0.37^c^	5.44 ± 0.84^a^	3.45 ± 0.16^e^	4.97 ± 0.44^b^	3.89 ± 0.11^d^	3.81 ± 0.28^d^
9	α-Terpinyl acetate	42.78 ± 2.19^c^	48.57 ± 1.60^a^	44.57 ± 3.61^b^	48.97 ± 2.09^a^	46.77 ± 3.64^ab^	48.67 ± 2.85^a^	42.37 ± 2.90^b^
10	Neryl acetate	24.33 ± 1.37^d^	18.48 ± 1.92^e^	23.35 ± 0.83^d^	31.48 ± 2.04^c^	33.45 ± 2.66^c^	42.67 ± 1.95^b^	49.88 ± 3.01^a^
11	(+/-)-*cis*-carveol acetate	0.84 ± 0.01^f^	0.91 ± 0.01^e^	0.72 ± 0.02^g^	0.97 ± 0.04^d^	1.31 ± 0.01^b^	1.67 ± 0.03^a^	1.06 ± 0.01^c^
Alcohols								
12	Linalool	429.48 ± 10.04^c^	387.92 ± 12.07^e^	402.48 ± 8.48^d^	450.97 ± 13.77^a^	438.77 ± 7.61^bc^	467.28 ± 11.32^ab^	442.70 ± 12.90^b^
13	Terpinen-4-ol	474.78 ± 12.07^b^	443.71 ± 10.45^d^	429.37 ± 9.61^e^	478.97 ± 8.69^b^	455.66 ± 11.37^c^	498.75 ± 5.94^a^	466.28 ± 10.08^c^
14	α-Terpineol	88.54 ± 5.74^c^	94.28 ± 6.05^bc^	78.79 ± 3.94^d^	96.57 ± 6.60^b^	84.08 ± 8.94^c^	103.83 ± 6.05^ab^	108.20 ± 8.63^a^
15	γ-Terpineol	164.59 ± 5.33^c^	169.74 ± 4.94^bc^	163.85 ± 7.28^c^	177.64 ± 5.66^a^	172.60 ± 4.31^b^	174.45 ± 4.20^ab^	170.54 ± 5.51^bc^
16	α-cadinol	12.97 ± 0.64^b^	11.04 ± 0.52^d^	12.43 ± 1.06^c^	9.71 ± 0.83^e^	14.67 ± 1.10^a^	10.87 ± 0.67^d^	13.87 ± 0.55^ab^
17	Carveol	0.57 ± 0.02^e^	0.65 ± 0.03^d^	1.26 ± 0.09^a^	0.87 ± 0.10^c^	1.03 ± 0.02^b^	1.24 ± 0.04^a^	0.65 ± 0.02^d^
18	γ-Eudesmol	29.48 ± 0.97^a^	25.45 ± 0.60^c^	26.84 ± 1.21^bc^	19.48 ± 0.97^e^	22.97 ± 1.77^d^	28.45 ± 1.34^ab^	27.32 ± 0.69^b^
19	Ledol	3.15 ± 0.14^e^	4.12 ± 0.09^d^	2.37 ± 0.07^f^	4.89 ± 0.34^b^	4.31 ± 0.28^c^	4.97 ± 0.32^ab^	5.22 ± 0.41^a^
20	L-α-Terpineol	125.82 ± 3.97^d^	138.54 ± 2.66^c^	144.23 ± 3.80^b^	154.21 ± 4.01^a^	140.65 ± 2.29^c^	148.11 ± 3.05^b^	139.98 ± 4.23^c^
21	β-Eudesmol	7.61 ± 1.07^b^	5.31 ± 1.35^e^	6.25 ± 2.08^d^	7.97 ± 1.05^b^	6.65 ± 0.94^c^	9.04 ± 1.52^a^	7.97 ± 2.34^b^
22	1-Octanol	1.94 ± 0.12^c^	2.97 ± 0.15^a^	1.27 ± 0.08^e^	1.67 ± 0.10^d^	1.88 ± 0.20^c^	2.24 ± 0.07^b^	2.56 ± 1.24^ab^
Aldehyde								
23	1-Nonanal	5.48 ± 0.98^c^	4.97 ± 0.24^d^	5.09 ± 0.61^d^	6.34 ± 0.44^b^	6.09 ± 0.83^bc^	6.93 ± 0.49^a^	5.95 ± 0.87^bc^
24	Octanal	0.65 ± 0.08^c^	0.75 ± 0.04^b^	0.84 ± 0.07^ab^	0.72 ± 0.06^b^	0.72 ± 0.11^b^	0.87 ± 0.13^ab^	0.91 ± 0.09^a^
25	(+)-Citronellal	8.03 ± 0.54^c^	10.29 ± 0.95^a^	6.05 ± 0.37^d^	9.21 ± 1.31^ab^	9.03 ± 1.06^b^	10.17 ± 0.99^a^	9.25 ± 1.20^ab^
Hydrocarbon								
26	3-Carene	164.87 ± 5.44^c^	150.04 ± 6.79^d^	162.04 ± 5.27^c^	169.23 ± 8.01^bc^	171.25 ± 4.35^bc^	182.01 ± 6.70^a^	175.59 ± 5.91^b^
27	(1r)-(+)-α-Pinene	195.49 ± 6.59^e^	221.34 ± 4.28^b^	209.57 ± 3.48^c^	216.34 ± 7.22^bc^	200.27 ± 5.30^d^	227.34 ± 7.55^b^	235.62 ± 4.26^a^
28	Sabinene	18.54 ± 1.34^c^	18.34 ± 1.69^c^	16.77 ± 1.55^d^	20.06 ± 1.30^b^	19.54 ± 2.07^b^	22.07 ± 3.13^a^	20.12 ± 1.67^b^
29	(1S)-(1)-β-Pinene	60.09 ± 3.05^e^	67.28 ± 1.21^c^	62.04 ± 2.94^d^	66.97 ± 3.33^bc^	71.03 ± 4.05^ab^	69.54 ± 3.20^b^	73.20 ± 2.97^a^
30	α-Phellandrene	13.65 ± 0.54^e^	16.97 ± 0.18^b^	12.30 ± 0.44^f^	15.44 ± 0.35^d^	13.07 ± 0.55^e^	16.64 ± 0.26^c^	17.82 ± 0.58^a^
31	α-Terpinene	465.44 ± 14.25^c^	450.34 ± 8.59^d^	446.08 ± 10.07^e^	475.61 ± 12.22^bc^	477.20 ± 11.18^b^	486.01 ± 9.94^a^	473.19 ± 13.21^c^
32	D-Limonene	1267.33 ± 21.24^d^	1287.01 ± 15.79^b^	1274.31 ± 27.62^c^	1275.34 ± 23.31^c^	1269.13 ± 16.68^d^	1312.75 ± 25.51^a^	1294.08 ± 20.67^ab^
33	γ-Terpinene	359.41 ± 11.48^cd^	368.97 ± 6.52^c^	352.77 ± 3.89^d^	375.12 ± 8.94^bc^	364.59 ± 7.66^c^	385.35 ± 9.89^a^	371.01 ± 10.05^c^
34	(4E,6Z)-Alloocimene	152.20 ± 3.27^d^	159.34 ± 5.88^c^	156.37 ± 4.31^c^	166.34 ± 2.94^b^	163.94 ± 3.95^bc^	169.97 ± 4.92^a^	167.58 ± 6.91^ab^
35	Copaene	3.34 ± 0.24^c^	3.64 ± 0.37^b^	3.84 ± 1.02^a^	2.97 ± 0.59^d^	3.13 ± 0.44^cd^	3.01 ± 0.72^bcd^	2.94 ± 0.61^d^
36	β-Myrcene	967.41 ± 15.40^bc^	977.10 ± 12.34^ab^	943.21 ± 17.60^d^	959.37 ± 9.58^c^	962.78 ± 12.05^bc^	984.75 ± 14.37^a^	971.34 ± 13.33^b^
37	α-Muurolene	2.46 ± 0.25^e^	2.68 ± 0.60^d^	3.65 ± 0.34^b^	4.29 ± 0.44^a^	2.77 ± 0.29^c^	4.09 ± 0.41^ab^	3.84 ± 0.38^b^
38	(−)-α-Cedrene	66.42 ± 4.30^d^	72.14 ± 2.91^c^	68.97 ± 6.73^cd^	79.27 ± 3.55^b^	68.04 ± 4.68^cd^	84.31 ± 6.04^a^	73.02 ± 7.58^bc^
39	(R)-γ-Cadinene	19.67 ± 2.01^d^	22.31 ± 1.67^bc^	20.76 ± 2.59^c^	26.84 ± 1.88^a^	22.01 ± 3.01^bc^	24.66 ± 1.86^b^	23.72 ± 2.35^b^
40	(+)-Δ-Cadinene	30.59 ± 3.87^c^	36.28 ± 4.60^ab^	31.04 ± 2.99^c^	35.45 ± 1.53^b^	36.75 ± 3.51^ab^	38.61 ± 2.85^a^	37.77 ± 1.66^ab^
41	Germacrene B	159.23 ± 7.64^c^	150.65 ± 6.55^d^	143.97 ± 4.97^de^	164.55 ± 8.61^b^	149.07 ± 9.08^e^	177.30 ± 7.33^a^	165.01 ± 8.20^b^
42	γ-Elemene	29.35 ± 2.07^e^	33.28 ± 1.86^c^	24.33 ± 2.22^f^	26.79 ± 0.94^d^	34.61 ± 1.30^b^	36.14 ± 1.08^a^	36.52 ± 2.11^a^
43	γ-Muurolene	12.86 ± 0.59^cd^	11.59 ± 0.73^d^	13.91 ± 0.41^b^	15.33 ± 0.34^a^	13.84 ± 0.62^b^	15.67 ± 0.55^a^	13.71 ± 0.76^b^
44	(Z)-β-Ocimene	367.87 ± 5.05^c^	351.01 ± 6.77^d^	345.97 ± 4.28^e^	378.04 ± 7.01^b^	367.45 ± 6.34^c^	388.20 ± 5.55^a^	369.46 ± 4.29^c^
45	(E)-β-Ocimene	754.65 ± 10.58^d^	772.00 ± 16.76^b^	762.19 ± 12.04^c^	760.08 ± 14.90^c^	770.31 ± 19.41^bc^	788.24 ± 16.00^a^	782.05 ± 15.97^ab^
46	β-Caryophyllene	68.55 ± 4.66^c^	57.31 ± 4.08^e^	56.94 ± 2.62^e^	74.64 ± 4.38^b^	60.28 ± 3.91^d^	82.33 ± 5.05^a^	76.31 ± 4.11^b^

Fig. 7(c) illustrates the regulation of aroma compound composition by different drying methods through variations in chord width and distribution. Terpenes such as β-myrcene, D-limonene, and α-terpinene exhibit extensive connections across multiple drying treatments; however, (US-SA)-(MFUS-FIRD) and (US-SA)-(MFUS-HAD) display markedly wider bands, indicating higher relative contributions of these compounds. Esters including linalyl acetate, geranyl acetate, and bornyl acetate form concentrated and broad chords under US-SA pretreatment, reflecting enhanced retention of high-boiling esters. Alcohols and terpenols (e.g., linalool, α-terpineol, γ-terpinol) also exhibit dominant wide chords under specific treatments, with linalool showing an especially broad connection zone, suggesting its pronounced proportion in the volatile profile. In contrast, single heat-source or atmospheric drying tends to induce cleavage, isomerization, and volatilization losses of esters and certain aldehydes, resulting in markedly narrowed chords in the diagram.

### 3.10 Electronic nose analysis

Radar plots and proportional distributions of electronic nose response values for *Zanthoxylum bungeanum* under different drying methods are shown in Fig. 9(a). All ten sensors responded to the dried samples, with particularly strong signals from W5S, W1S, W1W, and W2W, indicating higher concentrations of organic sulfides, terpenes, aromatic compounds, and nitrogen oxide compounds in dried *Zanthoxylum bungeanum*. The (US − SA)−(MFUS − FIRD) group exhibited the most pronounced response peaks, reaching 1.910, 27.736, and 5.670 on the key W3S, W1W, and W1S sensors, respectively, all markedly higher than those of MFUS − FIRD (0.984, 16.452, 4.044). This suggests that US − SA composite pretreatment mitigated oxidative polymerization and charring reactions, thereby retaining more abundant aromatic hydrocarbons, terpenes, and long − chain alkanes after drying. The aroma responses of (US − SA) − HAD were generally low, with W1W and W5S values of only 15.306 and 6.104, respectively, indicating oxidative degradation of terpenes and nitrogen oxides under high − temperature, oxygen − rich conditions and thus weakened aroma characteristics. The highest W5S value further reflected the accumulation of nitrogen oxides and thermal degradation products, consistent with prolonged thermo − oxidative exposure promoting oxidation of amines, pyridines, and certain amides. The responses of W1C, W5C, and W1W serve as indicators of the overall retention of aromatic hydrocarbons, aromatic compounds, and terpenes. Compared with (US − SA) − FIRD, W1C, W5C, and W1W values of (US − SA)−(MFUS − FIRD) increased by 19.92%, 10.79%, and 68.59%, respectively, demonstrating the advantage of the combined ultrasonic–thermal system in preserving aromatic constituents. Ultrasonic fields generate localized high pressure and shear disturbances at the microscale, inducing slight rupture of cell walls and oil gland vesicles and enhancing desorption and diffusion of aromatic compounds. Concurrently, ultrasonic microstreaming accelerates moisture migration and homogenizes heat distribution, reducing thermal cracking associated with localized overheating and thus improving the retention of aromatic hydrocarbons and terpenes. Li et al. [64], in their study on camellia oil, reported that ultrasonic treatment could effectively enhance the content of antioxidant-related metabolites in the material. The (US − SA) − MVD sample exhibited the highest W2W value (17.65), indicating the most pronounced release of aromatic and sulfur containing compounds. This is attributed to localized high pressure expansion induced by volumetric microwave heating, which triggers instantaneous release of encapsulated volatiles. However, its W1W value was only 15.60, significantly lower than (US − SA)−(MFUS − FIRD), indicating that excessively rapid temperature rise caused collapse of glandular structures in sample, leading to losses of terpenes, alcohols, and amides, and generating strong pungent and off − odor signals (high W3C and W5S responses). Notably, the effects of different drying methods on W2S and W3C responses were not significant (*P* > 0.05).

### 3.11 Microstructural analysis

**Fig. 8:**
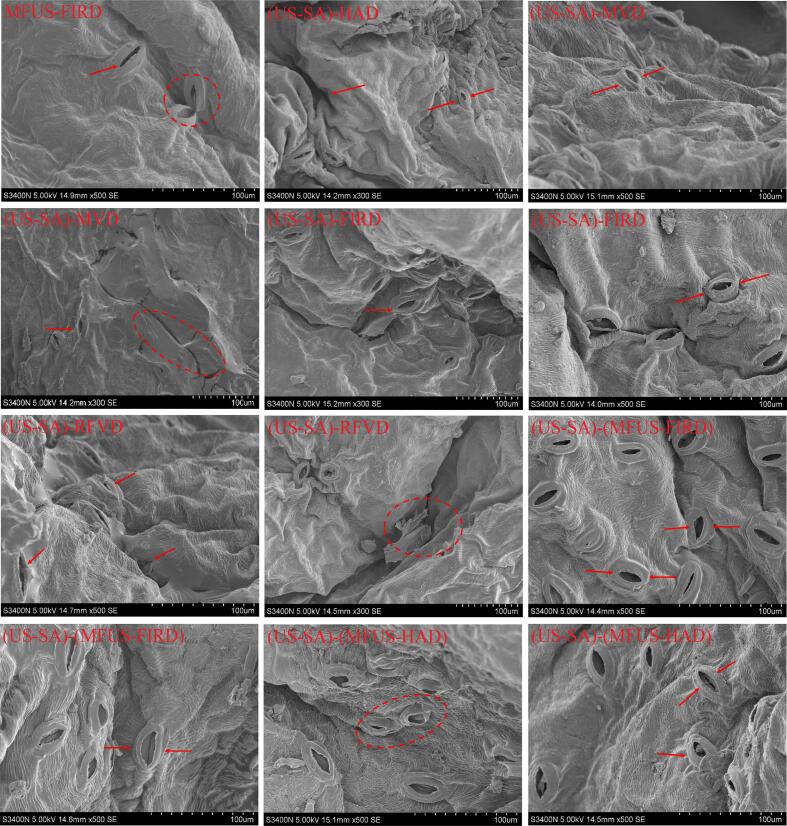
Effects of US–SA pretreatment combined with different drying processes on the microstructure of *Zanthoxylum bungeanum*.

SEM observations revealed pronounced differences in the microstructure of *Zanthoxylum bungeanum* pericarp after different drying treatments. Following HAD, the number of stomata markedly decreased and most became tightly closed; the surface tissue exhibited substantial densification, with the original porous architecture almost completely lost, accompanied by cracking, wrinkling, and deformation of the epidermis (Fig. 8). This results from the rapid evaporation of surface moisture under high − temperature airflow, which forms a rigid crust that hinders moisture outward diffusion, leading to severe internal stress accumulation and collapse of the cell wall. In samples subjected to MFUS − FIRD, cell walls remained largely intact, with abundant and uniformly distributed stomata that maintained a regular open state. Moreover, undulated folds were observed around stomatal cell walls, and the epidermal texture was clearly oriented, indicating shrinkage during drying while preserving overall structural continuity without apparent fracture or collapse. This suggests that moisture migrated uniformly through the porous channels, thereby preventing cell wall rupture induced by localized overheating or mass − transfer resistance. The synergistic action of MFUS improved water migration pathways, as acoustic cavitation and microstreaming enhanced moisture diffusion, whereas far − infrared vacuum heating provided uniform thermal input and established stable temperature and pressure gradients, collectively maintaining cell structural integrity. Ying et al. [65] similarly reported in a study on rhubarb that MFUS treatment expanded the internal microporous channels of the samples, thereby enhancing heat and mass transfer efficiency. After MFUS − HAD, the microstructure of the pericarp also showed relatively good integrity. As observed in Fig. 8, most cell walls retained tensile support, and stomata remained open and relatively abundant, although slight collapse and irregular shrinkage appeared in some regions, resulting in marginally reduced structural uniformity. This phenomenon is attributable to the limited heat transfer efficiency of HAD flow and rapid surface dehydration shrinkage. Nevertheless, the introduction of MFUS distinctly improved moisture migration channels and mitigated the severe collapse typically associated with HAD alone, allowing the overall microstructure to remain acceptable. In RFVD and MVD dried samples, the cell walls exhibited varying degrees of indentation, with fewer and irregularly shaped stomata; their overall structural support weakened but did not collapse entirely. This likely reflects the volumetric nature of electromagnetic heating, which induces rapid internal water evaporation within a short time, while nonuniform electromagnetic field distribution generates local hotspots that create excessive internal external pressure differentials, causing localized collapse or rupture of cell walls. Notably, the US − SA pretreatment exerted a protective effect on the microstructure of *Zanthoxylum bungeanum*. These results corresponded with Jansrimanee et al. [66]. As shown in Fig. 8, pretreatment substantially reduced cell wall collapse and yielded more regular and numerous stomata. Ultrasound − induced cavitation and micro − jetting increased cell wall permeability and facilitated moisture migration, whereas the SA coating formed a protective barrier that moderated pressure differences and delayed surface hardening, thereby suppressing cell wall collapse and stomatal closure. Zang et al. [67] investigated the effects of edible coating (SA) pretreatment combined with MFUS-assisted VFIR drying on the physicochemical properties of cherries. The results demonstrated that SA and ultrasound treatment contributed to a more intact internal tissue structure and promoted the formation of honeycomb-like porous networks within the cherries. These findings further confirmed the potential of the combined ultrasound − SA treatment in regulating microstructural characteristics, enhancing internal mass transfer pathways, and improving the overall drying quality of dried products.

### 3.12 Sensory properties

**Fig. 9:**
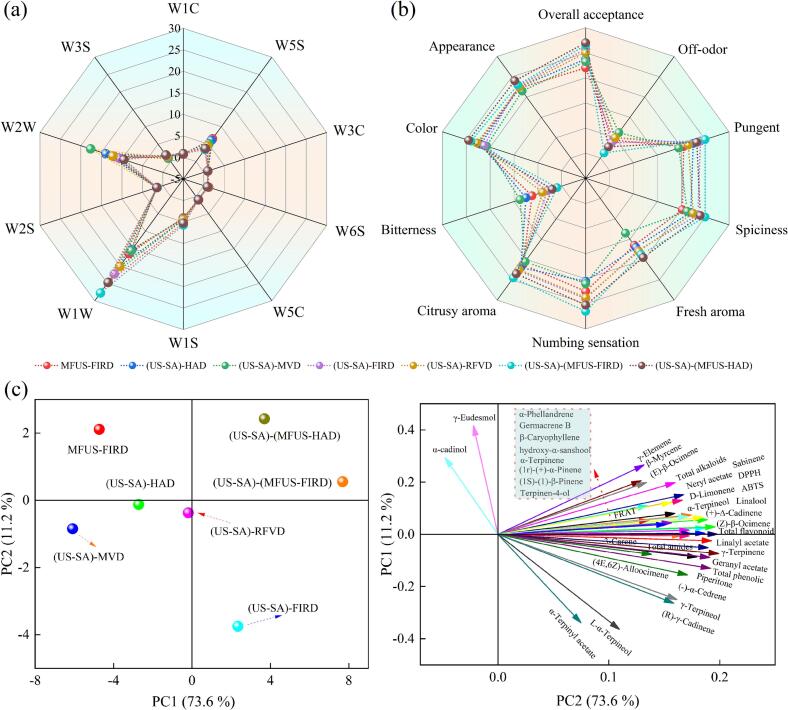
Electronic nose analysis, sensory properties, and PCA results of *Zanthoxylum bungeanum* after different drying treatments.

The sensory attributes of *Zanthoxylum bungeanum*, including taste, aroma, textural perception, and overall acceptability, constitute core indicators of its quality in seasoning and food applications, and these attributes vary markedly with drying method. The overall sensory scores for the seven drying treatments ranked as follows: (US − SA)−(MFUS − FIRD) > (US − SA)−(MFUS − HAD) > (US − SA) − FIRD > (US − SA) − RFVD > (US − SA) − HAD > MFUS − FIRD > (US − SA) − MVD (Fig. 9(b)). Among them, the (US − SA)−(MFUS − FIRD) samples exhibited the best performance across all indices, with the highest overall acceptance (8.8). Their microstructure showed well − supported cell walls and uniformly distributed pores, providing pathways for sustained release of tingling compounds, resulting in a persistent yet moderate numbing sensation while suppressing bitterness and off − odor. In terms of aroma, the fresh aroma, pungent aroma, and citrusy aroma were well preserved, with sensory scores of 5.5, 8.0, and 7.8, respectively, indicating that MFUS induced microperforation and energy dispersion effects mitigated thermal degradation of lipids and maintained the stability of aromatic compounds. The (US − SA)−(MFUS − HAD) samples ranked second in sensory quality, with an overall acceptance of 8.6. Their structure was substantially improved compared with (US − SA) − HAD, and the scores for numbing sensation, spiciness, and bitterness were 8.1, 7.6, and 0.8, respectively. This may be attributed to oxidative or thermal degradation of certain citrus like monoterpenes and aldehydes under localized high temperatures during HAD, while the energy dispersion effect of MFUS enhanced flavor retention and textural characteristics but could not fully prevent partial aroma loss. Compared with (US–SA)–(MFUS–FIRD), MFUS–FIRD samples showed reductions of 4.17%, 7.89%, 18.60%, 16.36%, and 22.50% in overall acceptance, color, numbing sensation, fresh aroma, and pungent aroma, while bitterness and off − odor increased by 3.13 and 1.59 fold. This resulted from the absence of US − SA pretreatment, which caused more severe cell wall damage, leading to excessive loss or oxidative degradation of certain flavor precursors and volatile aromatic compounds during drying. Meanwhile, localized heat accumulation intensified thermal reactions of proteins and lipids, generating bitterness and off − odor related compounds that weakened the persistence and complexity of fresh aromas, thereby diminishing overall sensory harmony. Notably, the (US − SA) − MVD samples recorded the highest bitterness score (3.5), primarily due to the localized overheating induced by the high energy density and nonuniform electromagnetic field in MVD, which promoted thermal degradation and oxidative transformation of polyphenols, sterol derived flavor precursors, and other constituents, generating quinone and aldehyde ketone compounds associated with bitterness. Luis et al. [68] similarly reported that electromagnetic heating heterogeneity and localized overheating in MVD can lead to flavor deterioration in the samples. Concurrently, the rapid drying stress caused collapse and shrinkage of cell structures, hindering the normal diffusion and release of volatile aromatic constituents. As a result, bitter compounds accumulated in intercellular spaces, further intensifying the perceived bitterness.

### 3.13 PCA analysis

Principal component analysis showed that PC1 (73.6%) and PC2 (11.2%) together accounted for 84.8% of the cumulative variance (Fig. 9(c)), indicating that the model adequately captured the overall quality differences among the drying treatments. PC1, representing the dominant axis of overall quality attributes, provided the strongest discrimination among treatments. As shown in the plot, the (US − SA)−(MFUS − FIRD) group was positioned at the far right of PC1 and exhibited clear spatial separation from the other treatments, indicating pronounced differences in its integrated quality characteristics. This suggests that the combined process exerted an enhancing effect across multiple quality dimensions, resulting in superior quality retention. Consistent results were also obtained from the correlation heatmap analysis (Fig. 7(b)). In contrast, (US − SA) − MVD was located at the far left of PC1, and this opposite distribution implies weaker performance in key quality attributes, likely related to localized overheating or degradation of active substances caused by the strong thermo–mass coupling during MVD. Along PC2, MFUS − FIRD and (US − SA)−(MFUS − HAD) appeared in the upper region of the score plot. Their pronounced positive shift along PC2 indicates distinctive characteristics in secondary yet discriminative quality variables. Conversely, (US − SA) − FIRD exhibited a clear negative deviation on PC2, further emphasizing its systematic difference from MFUS − FIRD. By comparison, (US − SA) − HAD and (US − SA) − RFVD were located near the center with minimal spatial separation between them, indicating that their performance across most quality indices was similar and did not exhibit the distinct shifts observed in other treatments. This distribution suggests that the two drying processes shared comparable physicochemical attributes and volatile profiles.

### 3.14 Correlation network heatmap analysis

**Fig. 10:**
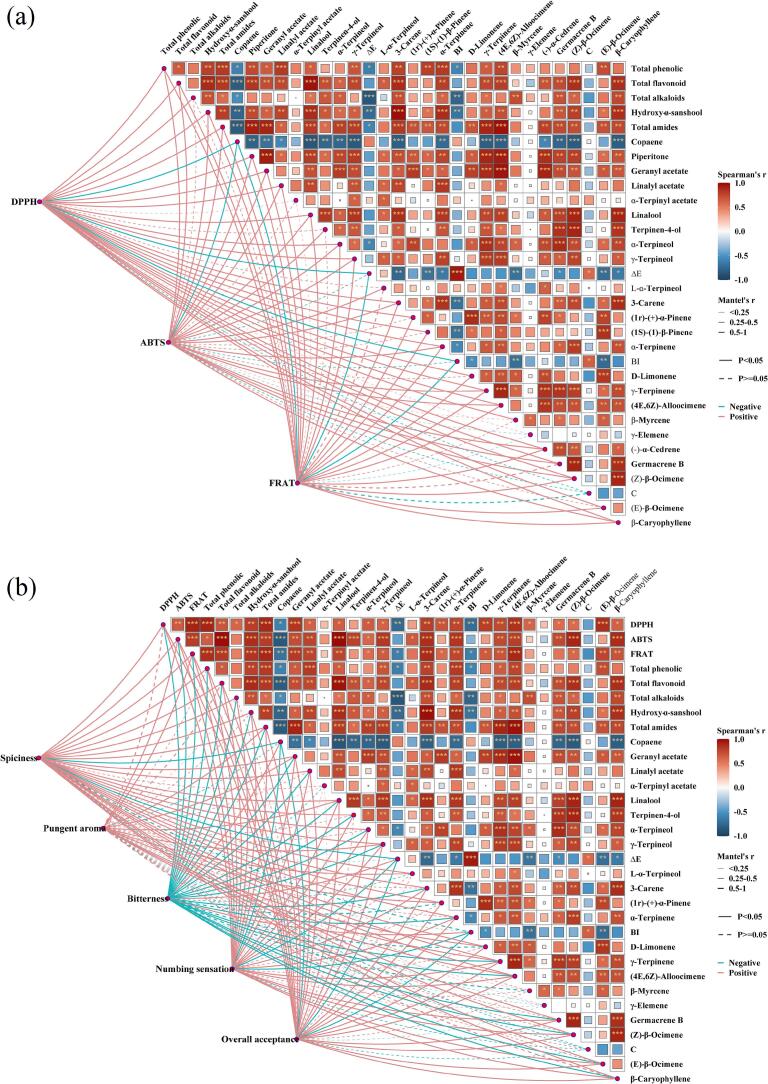
Correlation heatmap analysis of *Zanthoxylum bungeanum* after US–SA pretreatment combined with different drying processes.

In the antioxidant capacity network (Fig. 10(a)), DPPH, ABTS, and FRAP exhibited significant correlations with multiple chemical constituents. Total phenolics, total flavonoids, and hydroxy − α − sanshool formed a stable and strong positive correlation network with all three antioxidant indices, indicating that these compounds serve as the dominant chemical contributors to antioxidant capacity. Several terpenols (e.g., linalool, α − terpineol, terpinen − 4 − ol) also showed moderate positive correlations with the antioxidant metrics. The blue weak negative correlation blocks were mainly associated with certain monoterpene hydrocarbons (e.g., α − pinene, β − pinene), suggesting their limited antioxidant contribution or even a relatively antagonistic effect.

In the sensory attribute network (Fig. 10(b)), the tingling attribute was most closely correlated with hydroxy − α − sanshool and extended to several terpenols, indicating their dominant roles in eliciting the tingling and chemesthetic sensations, while the connections with terpenols further suggest that these compounds may influence overall perception by enhancing aroma complexity. The pungent attribute was strongly and positively associated with multiple monoterpenes and sesquiterpenes, demonstrating that the pungent sensation primarily derives from the coordinated release of terpene based volatiles. Bitterness showed weak negative correlations with several sesquiterpenes, implying that its formation mechanism may rely more heavily on nonvolatile constituents. Overall acceptability was positively associated with both terpenols and antioxidant indices, indicating that consumer preference is jointly driven by flavor pleasantness and the contribution of bioactive components, reflecting the combined influence of chemical aroma profiles and physiological attributes.

## 4 Conclusion

This study systematically evaluated the effects of US–SA pretreatment combined with multiple advanced drying technologies on the drying characteristics, energy consumption, and quality evolution of *Zanthoxylum bungeanum*. The results demonstrate that, although (US–SA)–MVD offers advantages in reducing drying time and energy consumption, its highly concentrated energy input and non–uniform heating readily induce severe structural damage, leading to pronounced losses of volatile flavor compounds and bioactive constituents, thereby limiting its applicability in processing highly volatile agricultural products. In contrast, samples treated with (US–SA)–(MFUS–FIRD) exhibited superior physicochemical quality and enhanced retention of key volatile compounds. These improvements are primarily attributable to the mitigation of cell wall collapse and stomatal closure by US–SA pretreatment, together with more homogeneous moisture migration and reduced localized thermal stress under (US–SA)–(MFUS–FIRD) conditions, which collectively enhanced the thermal stability and retention of terpenes and terpene alcohols. Correlation network analysis revealed that antioxidant capacity was predominantly driven by total phenolics, total flavonoids, and hydroxy-α-sanshool, whereas overall acceptance was determined by the synergistic contribution of volatile profiles and antioxidant performance. Moreover, sensory evaluation and PCA consistently confirmed that, relative to other dehydration methods, (US–SA)–(MFUS–FIRD) achieved the best comprehensive quality preservation and consumer acceptability.

Notably, the energy consumption of (US–SA)–(MFUS–FIRD) was 40.57 kW·h·kg^g−1^; future work should focus on optimizing the coupling mechanisms between multi–frequency acoustic field superposition and far-infrared heating to further improve process adaptability and industrial applicability. Nevertheless, several limitations should be acknowledged in the present study. First, although the quality evolution and physicochemical properties of *Zanthoxylum bungeanum* were systematically investigated, the underlying mechanisms governing energy transfer and acoustic–thermal coupling interactions at the molecular level have not yet been fully elucidated. Second, the experiments were conducted at the laboratory scale, and the long-term operational stability and industrial-scale feasibility of the proposed drying strategy still require further validation. Overall, the (US–SA)–(MFUS–FIRD) process provides a technically robust solution that reconciles quality preservation with energy efficiency for high–quality *Zanthoxylum bungeanum* processing, while offering a sound theoretical basis for the broader application of MFUS–assisted drying in agricultural and functional food systems.

## CRediT authorship contribution statement

**Zepeng Zang:** Writing – original draft, Software, Resources, Methodology, Investigation, Formal analysis, Data curation, Conceptualization. **Xiaopeng Huang:** Writing – review & editing, Visualization, Funding acquisition, Formal analysis, Conceptualization. **Guojun Ma:** Validation, Resources, Funding acquisition, Conceptualization. **Qiaozhu Zhao:** Software, Methodology, Data curation, Conceptualization. **Yanrui Xu:** Software, Methodology, Investigation, Data curation. **Bowen Wu:** Validation, Software, Data curation, Conceptualization. **Fangxin Wan:** Writing – review & editing, Resources, Project administration, Methodology, Formal analysis.

## Declaration of competing interest

The authors declare that they have no known competing financial interests or personal relationships that could have appeared to influence the work reported in this paper.
